# CEO narcissism and its influence on innovation capabilities of micro, small, and medium-sized enterprises: An empirical study in the Colombian context

**DOI:** 10.1371/journal.pone.0341491

**Published:** 2026-05-11

**Authors:** Tobías Alfonso Parodi Camaño, Jhon Víctor Vidal Durango, Ivan Portnoy

**Affiliations:** 1 Department of Industrial Engineering, Universidad de Córdoba, Colombia; 2 National Directorate of Research, Corporacion Unificada Nacional de Educacion Superior, Colombia; 3 Department of Chemistry, Universidad de Córdoba, Colombia; 4 Department of Productivity and Innovation, Universidad de la Costa, Colombia; Prague University of Economics and Business: Vysoka Skola Ekonomicka v Praze, CZECHIA

## Abstract

This study explores the relationship between the level of CEO narcissism and the innovation capabilities of firms in Colombia. Two structured instruments were applied to a sample of 85 companies from various economic sectors and organizational sizes. The first instrument measured narcissistic traits in CEOs based on an adaptation of the Narcissistic Personality Inventory (NPI), together with exploratory factor analysis (EFA). The second instrument assessed innovation capabilities across five key dimensions: product/service, internal processes, marketing, knowledge management, and investment in R&D. The results indicate significant associations between specific dimensions of narcissism and particular components of innovation, revealing both potential strengths and risks inherent to such traits. Theoretical and practical implications are discussed concerning the selection and training of executives in highly competitive environments.

## Introduction

In today’s dynamic business environment, characterized by technological disruption, intense competition, and constant uncertainty, the ability to innovate has become essential for the survival of any organization [1,2]. Within this context, the role of executive leadership has gained paramount importance, particularly in the design of strategies that enable firms to adapt, learn, and transform their operations. The Chief Executive Officer (CEO) is thus not merely an administrative authority but a true architect of the company’s strategic direction [3].

One of the individual traits that has captured the attention of organizational researchers is CEO narcissism. Defined as a combination of an inflated self-image, a constant need for admiration, and a strong desire for power, narcissism can drive leaders to pursue ambitious achievements and external visibility. However, it can also lead to egocentric decisions, excessive risk-taking, and pronounced resistance to criticism [4,5]. Interest in this trait has grown exponentially in recent years due to its potential influence on crucial strategic decisions, such as mergers, international expansion, technological change, and, most notably, innovation [6].

While narcissism is part of the broader set of dark triad personality traits, it differs conceptually from psychopathy and Machiavellianism [7,8]. Psychopathy is often linked to impulsiveness and antisocial tendencies, and Machiavellianism to strategic manipulation, whereas narcissism uniquely combines self-enhancement, a need for admiration, and leadership-oriented grandiosity. These features are closely aligned with high-visibility strategic initiatives and ambitious innovation efforts, which makes narcissism particularly relevant for examining innovation capabilities, as prior meta-analytic evidence indicates [9].

Various studies have shown that narcissistic CEOs tend to prioritize projects that generate high media impact, resulting in increased investments in research and development (R&D) and the adoption of bold strategies aimed at differentiating the company within its sector [6,10]. However, it has also been observed that these leaders may limit team participation, discourage dissent, and minimize others’ ideas. This, in turn, can negatively affect organizational learning processes and collaborative innovation [11,12]. This duality has fueled the need for empirical investigations analyzing the role of narcissism in shaping innovation capabilities, particularly in contexts where the CEO holds a significant concentration of power.

Despite the growing body of research in developed countries, there remains a significant gap in the literature regarding how this relationship manifests in emerging economies, especially within smaller firms. In countries such as Colombia, where micro and small enterprises constitute the backbone of the business landscape and leadership tends to be highly personalized, it is essential to understand how CEO traits can both foster and constrain innovation. This study, therefore, seeks to contribute to bridging this gap by integrating individual psychological variables with key organizational dimensions.

While previous research has examined the relationship between CEO personality traits and innovation capabilities through a sequential mediation framework [13], emphasizing the role of transformational leadership and innovative behavior as explanatory mechanisms, the present study adopts a fundamentally different perspective. Specifically, this research focuses on CEO narcissism as a multidimensional construct, disaggregating it into distinct facets (e.g., exhibitionism, leadership, and exploitativeness) and examining their differential and potentially opposing effects on multiple dimensions of innovation capabilities. Moreover, by incorporating interaction effects, this study highlights the configurational nature of CEO traits, showing that their impact on innovation is not purely additive but depends on specific combinations of characteristics. In this way, the present study complements prior work by moving beyond unified causal pathways and providing a more nuanced and heterogeneous understanding of how executive personality shapes organizational innovation outcomes.

### Research approach and study overview

This study adopts a quantitative, correlational, and cross-sectional research design, in line with the approaches most commonly used in research on executive leadership and organizational behavior [11,14]. This choice aligns with the study’s aim to examine significant associations between individual psychological traits and organizational strategic capabilities at a specific point in time, without manipulating variables or establishing direct causality.

To assess the level of CEO narcissism, an adaptation of the Narcissistic Personality Inventory (NPI) was employed. This is one of the most widely validated and commonly used scales in organizational contexts for identifying dimensions such as grandiose self-image, the desire for admiration, and self-referential leadership [9,14]. This instrument captures stable personality traits in leaders that may influence their decision-making styles.

Simultaneously, organizational innovation capabilities were evaluated through a structured questionnaire encompassing five fundamental dimensions: the development of new products and services, the improvement of internal processes, marketing innovation, knowledge management, and investment in research and development. This operationalization is grounded in the dynamic capabilities’ framework proposed by Teece [15] and has been applied in previous studies aimed at measuring organizations’ ability to adapt and generate value in changing environments [16,17].

Further data analysis was conducted using exploratory and confirmatory factor analyses, multifactor ANOVA and Pearson correlations to explore the strength and direction of the relationships between the various dimensions of narcissism and innovation capabilities.. This combination of methods enables the identification of relationships among variables while also indicating how strongly different narcissism traits relate to innovation capabilities. [12,18–20].

## Theoretical framework and hypotheses

### CEO narcissism and the upper echelons theory

The Upper Echelons Theory posits that organizations’ strategic decisions are, to a great extent, a reflection of the psychological characteristics, values, and experiences of their top executives [21,22]. Within this conceptual framework, narcissism has emerged as one of the most influential personality traits in shaping executive behavior. This trait is conceptualized as an inflated self-image, accompanied by a persistent need for admiration, a pronounced desire for power, and a tendency towards self-exaltation [23].

### Managerial narcissism and innovation propensity

It has been observed that CEOs with narcissistic traits tend to favor decisions aimed at generating media impact and strategic differentiation. This tendency may translate into higher levels of investment in innovation and a greater inclination toward high-risk projects [24]. Several empirical studies have documented a significant association between high levels of narcissism and innovative behaviors at the executive level. For example, Wang et al. [25] identified a curvilinear relationship between CEO narcissism and corporate innovation, suggesting that moderate levels of narcissism may be beneficial for innovation, whereas extreme levels tend to be dysfunctional.

Similarly, CEO narcissism positively influences technological exploration and the adoption of disruptive innovations, particularly in contexts characterized by high environmental uncertainty. This evidence reinforces the hypothesis that certain components of narcissism may act as catalysts for dynamic capabilities when managed within appropriate organizational structures [26].

### Negative implications of narcissism in leadership

Nevertheless, narcissism also entails significant risks. It has been documented that narcissistic CEOs exhibit lower openness to dissent, a tendency toward centralized control, and heightened sensitivity to criticism. Consequently, this can undermine collaboration, limit organizational learning, and create dysfunctional work environments [12,14,27]. Such limitations may hinder incremental innovation processes and negatively affect long-term organizational performance [10]. In summary, these findings reveal the ambivalent nature of narcissism in executive leadership: while it can foster bold, transformation-oriented strategic decisions, it may also compromise the relational and operational sustainability of organizations if not properly managed.

### Organizational innovation capabilities

Innovation capabilities are commonly conceptualized as the set of technological and organizational skills that enable a firm to transform ideas, knowledge, and resources into novel products, processes, and business models [17,28]. At the core of this construct lies the perspective of dynamic capabilities, defined by Teece et al. [29] as an organization’s ability to integrate, reconfigure, and direct its internal and external resources to respond effectively to changing environments.

Seminal research has demonstrated that this approach is fundamental not only for responding to competitive pressures but also for transforming them into sustainable advantages. For instance, Eisenhardt and Martin [30] are widely cited for showing that dynamic capabilities enable rapid and consistent reconfigurations of the organizational resource base, fostering adaptability and continuous innovation. Similarly, Zollo and Winter [31], in a highly cited analysis, highlight the importance of deliberate learning processes to ensure the effectiveness of these capabilities.

Additionally, systematic reviews, such as that conducted by Breznik and Hisrich [32], have confirmed that while dynamic capabilities and innovation capabilities are related, they present distinct conceptual differences that warrant separate analyses. This academic debate identifies innovation as a meta-capability that integrates areas such as strategic vision, competence base, organizational intelligence, creativity, systems, and corporate culture [33].

Operationally, innovation capabilities can be disaggregated into specific dimensions recognized for their strategic value: the ability to develop and launch new offerings to the market (product and service innovation); the continuous improvement and adoption of novel operational methods (internal process innovation); the exploration of new channels, messages, and marketing models (marketing innovation); knowledge management and innovation culture through the promotion of collaborative environments, collective learning, and the dissemination of ideas; and investment in research and development (R&D), which involves allocating financial and human resources to technological development, thereby enhancing the organization’s absorptive capacity and ability to leverage knowledge [34–36].

Particular attention has been given to small and medium-sized enterprises (SMEs) within emerging economies, where these capabilities are critical. For example, Deyassa [37] highlights that SMEs, despite their smaller scale, can achieve high levels of competitiveness through dynamic capabilities grounded in innovation, learning, and strategic alliances.

These dimensions enable the operationalization of the concept of organizational innovation and facilitate its measurement across different business contexts. Table 1 summarizes the key characteristics of each dimension, along with practical examples illustrating their application in real-world settings:

**Table 1:** Dimensions of organizational innovation capabilities (based on the work of Calik et al. [38]).

Dimension	Description	Application Examples
Product innovation	Focuses on the firm’s ability to create and improve products and services.	Launch of new products, design of customized digital services.
Process innovation	Relates to the ability to enhance and develop internal processes, manufacturing, and service delivery.	Automation of production processes, implementation of lean methodologies.
Marketing innovation	Pertains to the implementation of new marketing methods, including changes in product design, packaging, promotion, and distribution.	Innovative digital campaigns, use of non-traditional channels such as influencers or virtual reality (VR).
Organizational innovation	Refers to the firm’s ability to adopt new organizational methods, managerial processes, and structures.	Adopting agile management methods in production or service delivery to improve responsiveness.
Innovation culture	Represents the organizational culture that supports creativity, idea-sharing, and experimentation for innovation.	Reward systems for innovative ideas, such as employee suggestion programs with tangible incentives.
Innovation resources	Refers to tangible and intangible resources dedicated to innovation, such as R&D personnel, budgets, and technological assets	Allocating dedicated budgets for R&D to develop new products or technologies.

Consequently, innovation capabilities not only reflect a company’s ability to generate novel products or processes but also encompass the strategic vision, internal cohesion, and organizational culture that transform innovation into a sustained driver of competitive performance.

### Research hypotheses

Based on the reviewed literature and the proposed theoretical framework, the following hypotheses are formulated:

- **H1:** CEO narcissism is significantly associated with the firm’s innovation capabilities.

This general hypothesis is grounded in the assumption that CEOs’ personality traits, particularly narcissism, act as filters through which strategic decisions are processed [4,21]. Given its documented impact on bold initiatives and high-risk decisions, narcissism is expected to exert a broad influence on the organization’s innovation orientation [24,39].

- **H1a:** CEO narcissism positively influences product innovation.

Narcissistic leaders often seek visibility and recognition, which predisposes them to promote innovations that are visible to the market and consumers. Studies such as those by Wang et al. [25] and Liu et al. [22] have documented positive associations between narcissism and a focus on novel products, especially in competitive sectors.

- **H1b:** CEO narcissism positively influences investment in R&D.

A tendency toward risk-taking, combined with a need to stand out, may motivate narcissistic CEOs to allocate significant resources to technological or research projects that enhance their status both within and outside the [10].

These hypotheses reflect both the potential and the limitations of narcissism as a leadership trait. On the one hand, previous studies have shown that narcissistic CEOs tend to pursue visible and ambitious projects, which may promote innovations in products, services, or high-impact technological investments [4,6]. On the other hand, their inclination toward centralization and the need for external validation may undermine processes that require cooperation, active listening, and openness, such as knowledge-based collaborative innovation [11,12].

Moreover, it has been suggested that the effect of narcissism is not always direct but may operate as a moderator, either intensifying or weakening other key organizational relationships, such as the link between team autonomy and innovation capacity [40]. In this regard, this study seeks to examine not only linear effects but also more complex patterns of interaction between CEO traits and organizational structural factors, such as flexibility and organizational design.

## Methodology

### Research design

This study features a quantitative, correlational, and cross-sectional approach with the aim of analyzing the relationship between CEOs’ narcissistic traits and organizations’ innovation capabilities. The choice of design is justified by the need to measure psychological and organizational constructs through structured instruments that have been empirically validated in scientific literature, thereby allowing for the statistical assessment of their associations [41].

To measure narcissism, an adapted version of the Narcissistic Personality Inventory (NPI) was used. Originally developed by Raskin and Hall [42], the NPI has been widely applied and refined in subsequent research on personality within organizational contexts. This instrument captures key dimensions of narcissism such as authority, grandiosity, leadership, and the need for admiration.

For measuring innovation capabilities, the scale proposed by Calik et al. [38] was employed. This scale was developed and validated through a rigorous literature review and empirical testing with companies from various sectors. It encompasses five fundamental dimensions: product innovation, process innovation, marketing innovation, innovation culture, and resources for innovation. Its multidimensional structure and specific focus on small and medium-sized enterprises (SMEs) make it particularly relevant to the Colombian context of this study.

Both instruments were selected for their reliability, construct validity, and frequent use in peer-reviewed literature. Measurements were conducted using five-point Likert scales, which enabled the capture of subjective perceptions and the evaluation of underlying patterns in the relationships between the variables under study.

It is worth clarifying that this research does not aim to establish causal relationships between CEO narcissistic traits and firms’ innovation capabilities. Consistent with its quantitative, correlational, and cross-sectional design, the analyses are intended to identify statistically significant patterns of association rather than to infer directionality or causation. This methodology aligns with the exploratory nature of the study, particularly considering the limited literature for this research topic in emerging-economy MSMEs. By examining how specific dimensions of CEO narcissism relate to multiple organizational innovation capabilities, the study provides an initial empirical mapping of these relationships, offering a foundation for future longitudinal, experimental, or multilevel research designs that may address direct causal mechanisms.

### Population and sample

The target population consists of Colombian companies from various economic sectors. A non-probabilistic sample of 85 companies was obtained, identified through their participation in the innovation capabilities questionnaire. The CEOs that answered the questionnaires were recruited from April 11 to June 22, 2025. In accordance with ethical research standards and applicable Colombian data protection regulations, informed consent was obtained from all participants prior to their involvement in the study. Most of the organizations are based in the city of Montería, on the Colombian Caribbean, and primarily belong to the commerce sector. Moreover, all personal data were de-identified. In terms of size classification, the sample reflects a high proportion of microenterprises. Table 2 summarizes the sample’s main attributes.

**Table 2:** Sample attributes.

Variable	Most Frequent Category	Frequency
City	Montería	47
Position	Owner	33
Business Sector	Commerce	36
Size	Micro Enterprise	49

The sample comprised 85 companies, primarily located in Montería, a city that accounted for 55.3% of the responses. In total, organizations from 29 different cities across Colombia participated, suggesting a geographically diverse coverage, albeit with regional concentration. Regarding the positions held by participants, 38.8% indicated they were the owners of the company, followed by other administrative and managerial roles. This distribution ensures an adequate level of knowledge concerning strategic decision-making and innovation practices within the organizations.

About the economic sector, 28 different sectors were identified, with the commerce sector being the most represented, accounting for 42.4% of the responses. This diversity allows for the analysis of leadership influence within heterogeneous sectoral contexts. Concerning legal representation, all participants affirmed they were the legal representatives of their companies, validating the legitimacy of the informants in terms of authority and strategic knowledge.

Finally, regarding firm size, 57.6% of the organizations were classified as microenterprises, followed by small, medium, and large enterprises. This detail is relevant as it provides context for interpreting the results within the specific dynamics of micro and small enterprises, where the CEO or owner typically holds direct and centralized influence over decision-making processes.

### Instruments and theoretical basis

To measure the level of narcissism in CEOs, an adapted version of the Narcissistic Personality Inventory (NPI) was used. This instrument was originally developed by Raskin and Hall [42] and has been widely validated in subsequent research. It assesses multiple dimensions of narcissism, such as authority, exhibitionism, sense of superiority, vanity, exploitativeness, and leadership [43]. The NPI has proven to be reliable and applicable in organizational contexts for capturing narcissistic traits relevant to senior executives.

Organizational innovation capabilities were evaluated using a scale developed by Calik et al. [38], who proposed a multidimensional measurement model that has been empirically validated. This scale considers six key dimensions: product innovation, process innovation, organizational innovation, marketing innovation, innovation culture, and resources for innovation. The choice of this instrument is justified by its strong theoretical foundation and its prior validation in studies involving small and medium-sized enterprises (SMEs), making it particularly suitable for the context of this study.

The items included were perceptual and were answered using a five-point Likert scale, following the authors’ recommendations for capturing intangible constructs such as innovation [38]. This approach allows for an understanding not only of the visible outcomes of innovation but also of the internal capabilities that make it possible, a fundamental aspect in the analysis of emerging firms such as those in the sample of this study.

### Data retrieving

Data collection was conducted through an online questionnaire distributed via institutional email and professional networks. Participant confidentiality and the exclusive use of the data for academic purposes were guaranteed. Participation was voluntary, and informed consent was obtained in advance.

### Data analysis

Hereby, we provide a comprehensive description of the methods employed to retrieve the narcissism- and innovation-capabilities-related dimensions, as well as the further data analysis conducted with them. First, we addresse the confirmatory factor analysis (EFA) used to build the narcissism-related dimensions, as well as the further confirmatory factor analysis (CFA) to validate these constructs. Then, we describe the construction of the Innovation Capabilities-related dimensions and their validation via CFA. Finally, we show the analysis of variance (ANOVA) and the correlation analysis conducted to elucidate the underlying relationships between the narcissism- and Innovation Capabilities-related dimensions. All these analysis methods were implemented in R version 4.3.0 (2023-04-21 ucrt).

### Exploratory and confirmatory factor analysis of narcissism dimensions

As previously described, the NPI questionnaire comprises 40 questions or items with a 1−6 Likert scale, which are to make up the following dimensions (or factors): Authority, Exhibitionism, Superiority, Vanity, Exploitativeness, and Leadership. Some of these items are considered reverse pertaining to the exhibition of narcissism. Such items must be reversed by subtracting 7 from their raw sample-wise values and multiplying them by −1.

After the reverse items have been transformed, Bartlett’s test of sphericity must be applied to the data to confirm there are adequate relationships among these items to justify applying factor analysis [44]. Furthermore, the Kaiser-Meyer-Olkin (KMO) measure of sampling adequacy must be computed to assess the data’s suitability for factor analysis, i.e., to determine whether the patterns of correlations are compact enough to produce reliable and distinct factors [45,46].

Once the data suitability for factor analysis was confirmed by Bartlett’s test and the KMO metric, we proceeded with the EFA. For this purpose, we used the principal axis factoring (PAF) technique with the oblimin rotation [47,48]. This method reveals the underlying factor structure. For this EFA, we used the fa function within the R package psych [47]. As outlined by Calik et al. [38], we used six factors, aiming to find a match with those factors broadly reported in the literature. Subsequently, we screened out those scores (i.e., the measure of how individual items load to each constructed factor) below 0.4. Moreover, we further checked the cumulative variance of the structure matrix to assess whether it is high enough. This EFA finally reveals factor structure, i.e., the item-to-factor mapping to construct the six factors (or dimensions).

Nevertheless, prior to constructing the factors, we conducted a CFA with the factor structure revealed by EFA [49]. We used the cfa function within the R package lavaan [50], which is a kind of structural equation modeling (SEM) focused on testing whether a hypothesized factor structure (in this case, that yielded by the EFA) fits the observed data. As fitting metrics, we reported the comparative fit index (CFI), the Tucker-Lewis index (TLI), the root mean square error of approximation (RMSEA), and the standardized root mean square residual (SRMSR). Finally, we calculated the factor-wise Cronbach’s Alpha to evaluate how well each set of items (corresponding to each factor or dimension) combines to measure a single underlying construct [51].

When the factor structure has been unraveled and confirmed (with the EFA and CFA, respectively), the factors were computed by aggregating the items comprising each dimension using a column-wise arithmetic mean. This factor structure can be further used in the correlation analysis.

### Confirmatory factor analysis of innovation capabilities dimensions

The Innovation Capabilities questionnaire comprises 27 items on a 1–5 Likert scale. We used the item-to-dimension mapping outlined by Calik et al. [38]. This scale considers six key dimensions: product innovation, process innovation, organizational innovation, marketing innovation, innovation culture, and innovation resources. Nevertheless, we carried out a CFA to assess the fitness of this factor structure. Analogously as done with the narcissism questionnaire, we reported the CFI, TLI, RMSEA, and SRMSR fitting metrics. Also, we reported the factor-wise Cronbach’s Alpha (corresponding to each dimension).

### Analysis of variance and correlation analysis

With the narcissism- and innovation-capabilities-related factors previously constructed, we first conducted an ANOVA [52]. In this analysis, the narcissism dimensions are used as input variables, while the Innovation Capabilities dimensions are used as outcomes. Additionally, second-order interactions are assessed in order to unravel potential underlying interactions that a correlation analysis alone cannot reveal.

Furthermore, we computed the pair-wise Pearson correlation coefficient among all the dimensions pertaining to both constructs (i.e., narcissism and Innovation Capabilities). Also, we used Fisher’s Z test (with 95% confidence) to appraise the correlations’ significance [53]. This correlation analysis might reveal some dimension-wise interactions not pinpointed by the ANOVA, complementing it.

## Results

### EFA and CFA outcomes for the narcissism dimensions

Table 3 shows the item-to-factor mapping obtained from the EFA, along with a rationale for their labeling, and the Cronbach’s Alpha values for each factor.

**Table 3:** Narcissism exploratory factor analysis outcomes.

Factor	Retained Items	Notes	Cronbach’s Alpha
Authority	07, 13, 20, 30	Confidence, assertiveness, admiration-seeking	0.850
Exhibitionism	08, 10, 11	Showiness, needing attention, expressive behavior	0.750
Superiority	19, 29	Self-importance, deserving special treatment	0.912
Vanity	04, 05	Concern with physical appearance	0.564
Exploitativeness	17, 21, 33, 36	Manipulativeness, use of others for gain	0.785
Leadership	14, 18, 27	Desire for influence and control; blends social confidence and dominance	0.671

To assess the suitability of this factor structure, as mentioned in the Methodology Section (in Data Analysis), first, we computed Cronbach’s Alpha values (see Table 3), which support this premise. Then, we conducted Bartlett’s, which yielded a $\chi^{\text{2}}$value of 16862, which leads to a p-value considerably below 0.05 (near 0). Moreover, the KMO value resulted in 0.79, which indicates good adequacy for factor analysis. Subsequently, Table 4 presents the fitting metrics for the CFA, which overall endorse the factor structure obtained from the EFA.

**Table 4:** Narcissism confirmatory factor analysis fitting metrics.

Fit Index	Value	Interpretation
CFI	0.88	Near acceptable
TLI	0.847	Slightly low
RMSEA	0.089	Acceptable
SRMR	0.074	Good

### CFA outcomes for innovation capabilities dimensions

Table 5 illustrates the factor structure for the innovation capabilities questionnaire, as outlined by Calik et al. [38], along with Cronbach’s Alpha values, while Table 6 presents the resulting CFA fitting metrics.

**Table 5:** Innovation capabilities item-to-factor mapping and Cronbach’s Alpha values.

Factor	Items	Cronbach’s Alpha
Product Innovation	Prod1, Prod3, Prod4, Prod5	0.724
Process Innovation	Proc2, Proc3	0.596
Organizational Innovation	Org1, Org2, Org3	0.734
Marketing Innovation	Mark1–Mark4	0.789
Innovation Culture	Cult2–Cult5	0.812
Resources for Innovation	Res1–Res8	0.893

**Table 6:** Innovation capabilities confirmatory factor analysis fitting metrics.

Fit Index	Value	Interpretation
CFI	0.797	Marginal fit
TLI	0.765	Marginal fit
RMSEA	0.114	Poor fit
SRMR	0.081	Acceptable

Overall, in light of the CFA outcomes, the factor structure proposed by Calik et al. [38] is acceptable and can be used for further analysis.

### ANOVA and correlation analysis outcomes

**Fig 1:**
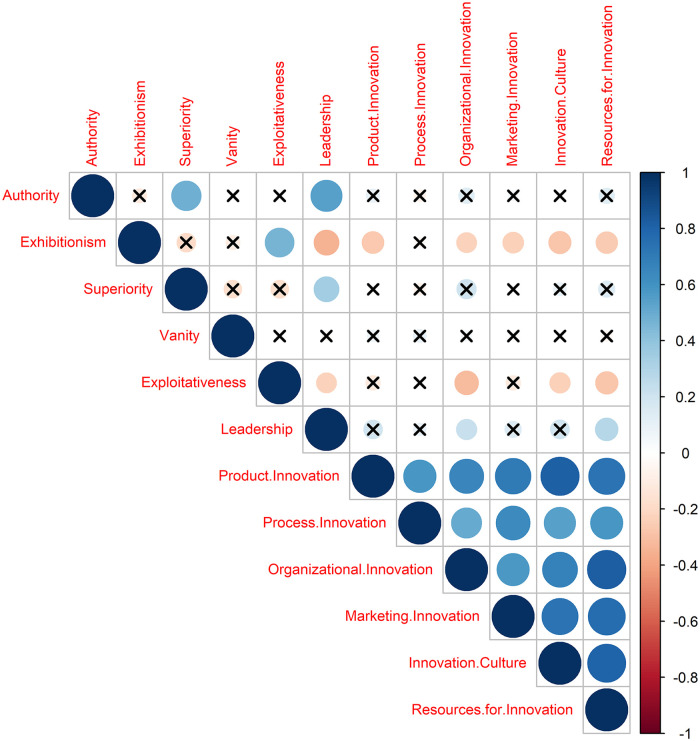
Dimension-wise correlation structure.

Fig 1 shows the (Pearson) pair-wise correlations for the narcissism and innovation capabilities dimensions. In this heatmap, the correlation matrix is visualized through circles whose size and color reflect the strength and direction of the relationships between variables. Blue circles indicate positive correlations, and red circles indicate negative correlations. The larger and darker the circle, the stronger the correlation. Crosses denote non-significant correlations. Notice that the correlation structure cannot reveal second-order interaction inputs. Thus, we conducted an ANOVA to assess the impact of all narcissism-related dimensions and their second-order interactions on the innovation capabilities-related dimensions.

Table 7 summarizes both the significant pair-wise correlations between narcissism-related dimensions and innovation capabilities-related dimensions and the significant effects found by the ANOVA. Additionally, Table 7 presents the correlation values, significance status, and direction (positive or negative), along with the ANOVA’s p-values and η² values to assess the interactions’ effect sizes.

**Table 7:** Correlation analysis and ANOVA outcomes summary.

Narcissism Factor/Interaction	Innovation Outcome	Correlation/ Significant?/ Direction	ANOVA p/ η²	Interpretation
Exhibitionism	Product Innovation	−0.263/ Significant/ Negative	p = 0.024/ η² = 0.08	Strong agreement
	Marketing Innovation	−0.239/ Significant/ Negative	p = 0.046/ η² = 0.06	Strong agreement
	Organizational Innovation	−0.221/ Significant/ Negative	p = 0.06/ η² = 0.06	Moderate agreement
	Innovation Culture	−0.272/ Significant/ Negative	p = 0.014/ η² = 0.09	Strong agreement
	Resources for Innovation	−0.259/ Significant/ Negative	p = 0.028/ η² = 0.07	Strong agreement
Exploitativeness	Organizational Innovation	−0.312/ Not significant/ Negative	p = 0.038/ η² = 0.07	Added insight from ANOVA
	Innovation Culture	−0.230/ Significant/ Negative	p = 0.314/ η² = 0.02	Added insight from corr. analysis
	Resources for Innovation	−0.279/ Significant/ Negative	p = 0.117/ η² = 0.04	Added insight from corr. analysis
Leadership	Organizational Innovation	0.229/ Significant/ Positive	p = 0.366/ η² = 0.01	Added insight from corr. analysis
	Resources for Innovation	0.287/ Significant/ Positive	p = 0.155/ η² = 0.03	Added insight from corr. analysis
Exhibitionism × Leadership	Innovation Culture	NA	p = 0.0318/ η² = 0.07	Added insight from ANOVA

Note. η² indicates the effect size in the ANOVA models and represents the proportion of variance in the innovation capability dimension explained by the corresponding narcissism factor or interaction.

From Table 7, the findings reveal that Exhibitionism emerges as the strongest dimension with effects on various innovation outcomes, including Product Innovation, Marketing Innovation, Innovation Culture, and Resources for Innovation. This is evidenced both by its significant Pearson correlations and its moderate effect sizes in the ANOVA models. Furthermore, the ANOVA results highlight interaction effects (particularly between Exhibitionism and Leadership) that were not apparent in the correlation analysis, suggesting that the impact of narcissistic traits on innovation is not purely additive but may depend on specific combinations of traits.

Notably, Exploitativeness and Leadership also exhibit strong relationships with some innovation-related outcomes. Overall, these results suggest that while Exhibitionism alone plays a central role, the interplay between narcissistic dimensions adds nuance to understanding how personality traits relate to different aspects of innovation.

### Hypothesis testing and empirical tests

To validate the hypotheses formulated in the theoretical framework, a joint analysis of the empirical findings was conducted. Table 8 summarizes the degree of empirical support for each of the proposed hypotheses:

**Table 8:** Contrasting hypotheses based on empirical results.

Hypothesis	Description	Evidence Found	Outcome
H1	CEO narcissism is significantly associated with innovation capabilities.	Moderate but significant correlations and effects (from ANOVA) between narcissism dimensions and innovation variables.	Confirmed
H1a	CEO narcissism positively influences product innovation.	Negative correlations and moderate but significant (ANOVA) effects between dimensions of CEO narcissism and the Product Innovation dimension.	Falsified
H1b	CEO narcissism positively influences R&D investment.	Moderate but significant correlations and effects (from ANOVA) between dimensions of CEO narcissism and the Resources for Innovation dimension. Some correlations are positive and some negative.	Partially confirmed

As can be observed, hypotheses H1 and H1b receive partial support based on the correlation and regression analyses, while hypothesis H1a has been falsified with our data.

## Discussion

Based on the results obtained, the set of hypotheses formulated in the theoretical framework is contrasted below. The general hypothesis (H1), which proposed a significant association between CEO narcissism and innovation capabilities, was partially confirmed. Significant correlations were observed between certain dimensions of narcissism (Exhibitionism, Exploitativeness, and Leadership) and specific aspects of innovation (Product, Marketing, and Organizational Innovation, Innovation Culture, and Resources for Innovation), as well as mild, significant effects revealed by the ANOVA. These findings support previous studies that identify narcissism as an ambivalent trait, capable of stimulating bold and innovative behaviors, but also of generating strategic risks [18,54].

Hypothesis H1a, which posited a positive influence of narcissism on product innovation, was falsified, as mild (yet significant) negative correlations were identified between this innovation dimension and the exhibitionism dimension of narcissism. Moreover, the ANOVA revealed a mild, significant effect of the exhibitionism dimension on product innovation as well. In contrast, H1b, related to the positive influence on R&D investment, was partially empirically supported through a moderate positive correlation with the leadership dimension and the resources for innovation dimension. However, the latter showed (significant) negative correlations with the exhibitionism and exploitativeness dimensions, hence the partial nature of this hypothesis confirmation.

The findings allow for interpreting the role of CEO narcissism as an ambivalent phenomenon. On the one hand, the outcomes suggest that certain narcissistic traits can act as drivers of innovation, fostering bold decisions, organizational visibility, and strategic ambition. This aligns with the arguments of Chatterjee and Hambrick [23], who contend that narcissistic CEOs tend to favor initiatives that increase their notoriety, such as disruptive innovation or high-profile projects. Our results align with the thesis that the impact of narcissism is neither homogeneous nor deterministic. Previous studies have also indicated that these effects may vary depending on the competitive environment and the type of innovation considered [11,12].

From a practical perspective, the findings highlight the importance of identifying the most suitable leadership profile according to the organizational context. Recognizing the potential strategic benefits of certain narcissistic traits does not imply overlooking their risks, especially in environments with low institutional control. Organizations could benefit from coaching and development processes that channel these tendencies toward sustainable innovative goals without compromising organizational cohesion.

Taken together, the findings underscore the importance of adopting a nuanced view of narcissistic CEOs, recognizing both their strategic potential and their latent risks. Far from advocating a one-dimensional assessment of narcissism, this study invites a critical reflection on how certain traits can be positively channeled, provided that appropriate organizational conditions are in place.

To further clarify this nuanced picture, it is useful to consider the differential effects of narcissism facets, which can be better understood by considering their underlying psychological mechanisms. Exhibitionism reflects attention-seeking and self-promotional tendencies [55], which may lead leaders to overestimate their own creativity while channeling fewer resources to sustained, collaborative innovation processes. In contrast, authority and leadership-oriented facets are linked to dominance, self-confidence, and greater tolerance for risk, characteristics that may support bold strategic initiatives and willingness to invest in innovation [56]. Conversely, exploitativeness, marked by low empathy and an instrumental view of others, may weaken trust, knowledge sharing, and collective learning [57]. These distinctions highlight that narcissism does not operate as a uniform trait but influences organizational outcomes through multiple psychological pathways.

Moreover, it is worth considering that the predominance of centralized decision-making in Colombian MSMEs may also be interpreted through the lens of cultural characteristics such as power distance. In organizational contexts where hierarchical authority is more readily accepted and upward dissent is limited, CEO personality traits may exert a stronger influence on strategic outcomes. This suggests that narcissistic tendencies, whether expressed through bold strategic vision or reduced openness to disagreement, may be amplified in such environments, reinforcing the importance of considering cultural and structural factors when interpreting the observed relationships.

Finally, this study presents limitations that should be acknowledged. First, the cross-sectional and associational design does not allow causal inferences, and the findings should be interpreted as relational patterns rather than directional effects. Second, the sample size, while comparable to many studies involving CEOs and SMEs in developing economies, is modest for SEM-based techniques. Confirmatory factor analysis was used primarily for measurement validation, and model fit indices may be sensitive to sample size constraints. Additionally, the use of purposive and convenience sampling, necessary due to the practical challenges of accessing MSMEs, may limit the generalizability of the findings. Moreover, because the sample includes firms of different sizes, and CEO influence is typically stronger in smaller and less formalized organizations, the predominance of microenterprises in our sample may condition the magnitude of the observed relationships. Future studies could examine size-based differences through multi-group analyses, as the sample size makes it prohibitive for this research.

Yet another limitation arises due to the use of self-report measures for both CEO personality traits and innovation capabilities. Narcissistic individuals are prone to displaying self-enhancement biases, which may influence not only their self-assessment but also their appraisal of organizational characteristics. Thus, the observed associations may reflect relationships between perceived CEO personality and perceived innovation capabilities rather than directly observed innovation outcomes. Future research could address this issue by incorporating multi-source data, such as employee reports and historical innovation (quantitative) indicators, to reduce common method and perceptual bias.

Despite these constraints, the study provides an initial empirical appraisal of how specific CEO personality traits relate to multiple innovation capabilities in emerging-economy MSMEs. Future studies with larger, longitudinal, or multilevel designs could further examine causal mechanisms and validate the measurement structure across diverse settings.

Another future research line should study the potential nonlinear nature of the relationship between narcissism and innovation. Recent studies suggest that moderate levels of narcissistic traits may be associated with strategic boldness and visionary leadership that support innovation, while high levels may hinder collaboration, learning, and long-term effectiveness [58].

## Conclusions

This study provides an empirical exploration of how CEO narcissism relates to innovation capabilities in Colombian firms. The findings suggest that certain facets of narcissism, such as a strong leadership orientation, are positively linked to key innovation practices, particularly investment in research and development (R&D). However, it is crucial to interpret these connections with caution, as they do not constitute causal relationships, but rather relational patterns derived from self-report measures. This observation aligns with recent literature, which warns of the ambivalent nature of narcissistic traits, capable of driving bold visions but also leading to impulsive decisions.

From a theoretical perspective, these results not only support but also enrich the Upper Echelons Theory. They underscore how CEOs’ personality traits can be a determining factor in shaping organizational dynamics as vital as innovation. On a practical level, the findings highlight the pressing need to strategically assess and manage these traits during leadership selection and development processes. It is imperative to foster a delicate balance between individual ambition and the collective sustainability of the firm.

A distinctive contribution of this research lies in its contextual focus on Colombia, where micro, small, and medium-sized enterprises (MSMEs) account for more than 90% of the business landscape, serve as key pillars of employment, and often operate under highly centralized power structures. According to national (Colombian) classification, a microenterprise has up to 10 employees; a small enterprise, up to 50; and a medium-sized enterprise, up to 200. This configuration makes the role of the CEO even more significant, as their decisions tend to exert direct influence with little intermediation within the organizational structure.

Moreover, the combination of structured instruments and multivariate statistical analyses lends significant methodological rigor to a field largely dominated by qualitative or exploratory studies. Nevertheless, it is important to acknowledge the limitations of this study, inherent to its cross-sectional design, the use of self-reports, and sample size. These limitations constrain the generalizability of the results, although they are common in research at the executive level. Therefore, the adoption of complementary methodological strategies, such as longitudinal designs, multilevel analyses, and multi-source data, is emphasized.

For future research, it would be valuable to incorporate analyses segmented by business sectors and firm size, expand the sample to various regions of the country, and consider organizational environments with distinct cultural characteristics. It would be particularly enlightening to contrast these results with international samples that reflect different cultural, institutional, and economic realities. This would allow for assessing whether the observed effects of CEO narcissism on innovation possess a universal character or are context-dependent.

Finally, continued research into the interaction between executive personality and innovation is encouraged. It is also crucial to consider the competitive environment and the type of innovation (incremental vs. disruptive), as recent studies in the field of strategic psychology have proposed.

## Supporting information

S1 File The supporting files available at https://zenodo.org/records/18459757 include: Inn_Data.csv, which contains the answers to the Organizational Innovation Capabilities Questionnaire. npi40_responses.csv, which contains the answers to the Narcissistic Personality Inventory (NPI) Questionnaire. Negative.csv, which shows, for each question of the NPI questionnaire, whether it is negative (and therefore should be inverted) or not Narcissism and Innovation Capabilities Source Code.R, which contains the source code in R (version 4.3.0 (2023-04-21 ucrt)) used to conduct the analysis methods version 4.3.0 (2023-04-21 ucrt).
(ZIP)
